# Retinal organoids and microfluidic chip-based approaches to explore the retinitis pigmentosa with USH2A mutations

**DOI:** 10.3389/fbioe.2022.939774

**Published:** 2022-09-14

**Authors:** Ting Su, Liying Liang, Lan Zhang, Jianing Wang, Luyin Chen, Caiying Su, Jixing Cao, Quan Yu, Shuai Deng, Hon Fai Chan, Shibo Tang, Yonglong Guo, Jiansu Chen

**Affiliations:** ^1^ Department of Ophthalmology, First Affiliated Hospital of Jinan University, Jinan University, Guangzhou, China; ^2^ Key Laboratory for Regenerative Medicine, Ministry of Education, Jinan University, Guangzhou, China; ^3^ Centric Laboratory, Medical College, Jinan University, Guangzhou, China; ^4^ Institute for Tissue Engineering and Regenerative Medicine, Chinese University of Hong Kong, Hong Kong, China; ^5^ Key Laboratory for Regenerative Medicine of the Ministry of Education of China, Ministry of Education of China, School of Biomedical Sciences, Faculty of Medicine, Chinese University of Hong Kong, Hong Kong, China; ^6^ Aier Eye Institute, Changsha, China; ^7^ College of Veterinary Medicine, South China Agricultural University, Guangzhou, China; ^8^ Institute of Ophthalmology, Medical College, Jinan University, Guangzhou, China

**Keywords:** retinitis pigmentosa, USH2A, induced pluripoten stem cells, retinal organoids, microfludic chip

## Abstract

Retinitis pigmentosa (RP) is a leading cause of vision impairment and blindness worldwide, with limited medical treatment options. USH2A mutations are one of the most common causes of non-syndromic RP. In this study, we developed retinal organoids (ROs) and retinal pigment epithelium (RPE) cells from induced pluripotent stem cells (iPSCs) of RP patient to establish a sustainable *in vitro* RP disease model. RT-qPCR, western blot, and immunofluorescent staining assessments showed that USH2A mutations induced apoptosis of iPSCs and ROs, and deficiency of the extracellular matrix (ECM) components. Transcriptomics and proteomics findings suggested that abnormal ECM-receptor interactions could result in apoptosis of ROs with USH2A mutations via the PI3K-Akt pathway. To optimize the culture conditions of ROs, we fabricated a microfluidic chip to co-culture the ROs with RPE cells. Our results showed that this perfusion system could efficiently improve the survival rate of ROs. Further, ECM components such as laminin and collagen IV of ROs in the RP group were upregulated compared with those maintained in static culture. These findings illustrate the potential of microfluidic chip combined with ROs technology in disease modelling for RP.

## 1 Introduction

Retinitis pigmentosa (RP), a group of heterogeneous inherited retinopathy, is characterized by early-onset of nyctalopia, progressive visual field defects and irreversible blindness. The worldwide prevalence of RP is estimated at approximately 1 in 4000 (Verbakel et al., 2018). Despite recent innovative advancements in retinal therapeutics, such as gene and cellular therapies, there are currently no effective treatments to prevent or slow the progression of RP (Botto et al., 2022). USH2A gene mutations are the leading cause of autosomal recessive non-syndromic RP, accounting for 12–25% of all RP cases (Toualbi et al., 2020). To date, more than 1100 disease-causing variants in USH2A, including nonsense and missense mutations, splicing variants, small in-frame insertions and deletions, have been identified (Gao et al., 2021). Among them, c.8559–2A>G is the hotspot mutation in the Asian population (Nakanishi et al., 2011).

The USH2A gene encodes a large protein called usherin which contains laminin epidermal growth factor and fibronectin type III motifs. These motifs have been most commonly observed in the protein components of the basal laminin, extracellular matrixes, and cell adhesion molecules (Bhattacharya and Cosgrove, 2005). Genetic *in vivo* studies have shown that usherin is essential for the survival of photoreceptor cells and inner ear hair cell bundle integrity (Zou et al., 2011; Perrino et al., 2021; Schwaller et al., 2021). Although these studies above provide clues for uncovering the functions of the USH2A gene, however, its large size and broad spectrum of mutations raise many challenges for researchers to develop effective gene therapy (Stemerdink et al., 2021). Further, even though animal models could recapitulate RP phenotypes and have demonstrated promising prospects for developing therapies, photoreceptor degeneration in such model was found to be very slow and could not be observed until at least 10 months of age (Lu et al., 2010; Sundar et al., 2020). In addition, although retinal explants from human donors can provide a full-featured model to investigate the pathogenesis of RP, obtaining fresh human tissues in clinical settings is challenging and seems impractical.

Over the last decade, the advent of induced pluripotent stem cells (iPSCs) has revolutionized the utility of human *in vitro* models (Achberger et al., 2019; Kim et al., 2020). With this technology, retinal organoids (ROs) could be established from RP patients and have been used to study the underlying mechanisms for researching novel therapies (Lancaster and Knoblich, 2014; Zhong et al., 2014; Deng et al., 2018). Several groups have performed functional studies using iPSC-derived organoids to model various aspects of ocular diseases and have provided concrete evidence showing that organoids were capable of recapitulating certain well-known pathological processes of PR (Liu et al., 2020; VanderWall et al., 2020). Huang et al. generated a valid three-dimensional *in vitro* RO model that could replicate the key features of X-linked juvenile retinoschisis, including retinal splitting and defective retinoschisin production from patients with RS1 gene mutation (Huang et al., 2019). Tucker et al. successfully produced three-dimension (3D) eye cup-like structures derived from a patient carrying USH2A mutations to illustrate that endoplasmic reticulum (ER) stress was involved in USH2A-related pathogenesis (Tucker et al., 2013). In our previous study, we showed that ROs generated from the iPSCs of a patient with USH2A mutations exhibited early developmental abnormalities (Guo et al., 2019). Further, transcriptomic analysis demonstrated that the ROs possessed developmental similarities with native human retina (Cui et al., 2020). Together, these suggest that retinal organoids are of utmost importance for RP research.

Currently, the main concern of 3D retinal organoid models is that as they grow in size and volume, oxygen and nutrient availability to the growing cells becomes limited and leads to the build-up of a necrotic core (Yu et al., 2019). To solve this conundrum, microfluidic cell culture, which can mimic the cellular microenvironment, has been developed, and offers several advantages over traditional cell culture (Park et al., 2019). First, microfluidic systems allow precise control of fluids, nutrients delivery, and washout of the cultured cells. Second, they could actualize cell-cell crosstalk. Finally, microfluidic devices allow researchers to customize a physiological niche-like “nichoid” microenvironment resembling *in vivo* conditions (Hofer and Lutolf, 2021; Messa et al., 2021).

Bioreactors have been shown to improve the laminar stratification and yield of photoreceptor cells bearing cilia and nascent outer-segment-like structures (Ovando-Roche et al., 2018). Thus, they represent a promising platform for scaling up the production of retinal organoids. In 2019, Achberger et al. were the first to introduce the retina microfluidic chip, also called retina-on-a-chip, which successfully imitated the physiological interactions of mature photoreceptor segments with retinal pigment epithelium (RPE) *in vitro*. The chip possessed vasculature-like perfusion for precisely controllable delivery of stimulants and physiological secretion kinetics (Achberger et al., 2019). Peak et al. reconstructed the outer blood-retinal barrier (oBRB) by co-culturing iPSC-derived RPE cells with vascular endothelial cells on a microphysiological engineering chip. The engineered oBRB demonstrated by a well-defined network of intercellular tight junctions replicate the relative spatial arrangement of the RPE cells in a more realistic manner that conventional models based on Transwell inserts (Paek et al., 2019). Therefore, retina microfluidic chips seem to be able to allow organoids differentiation and maintenance.

In this current study, we established patient-specific iPSC cell lines with USH2A mutations (c.8559–2A>G and c.9127_9129delTCC) (KLRMMEi001-A), followed by ROs and RPE differentiation from the iPSCs to create an *in vitro* RP disease model. To elucidate the molecular mechanism of RP, transcriptomic and proteomic analyses were performed to identify significantly regulated genes and proteins related to USH2A mutations. Further, an iPSCs line named XT142-iPS with USH2A gene mutation (only c.8559–2A>G) (KLRMMEi002-A) from a USH2 patient was created and used for ROs differentiation. We also constructed a microfluidic chip to co-culture ROs and RPE cells with USH2A mutations, as advancement to current organoid models.

## 2 Material and methods

### 2.1 Generation of ROs and RPE from iPSCs

To generate ROs and RPE from iPSCs for pathophysiologic studies, urine samples were obtained from a 27-year-old male patient diagnosed with RP. Informed written consent was obtained from the patient and the experiments were performed following the Declaration of Helsinki. This study protocol was approved by the Ethics Review Board of Jinan University (Guangzhou, China). The iPSCs (RPiPSCs) were produced by urine cells as previously described (Guo et al., 2018) and the XT142-iPSCs were created using peripheral blood mononuclear cells (Liang et al., 2022). To maintain pluripotency, iPSCs were cultured in an mTeSR1 medium (Stemcell Technologies). ROs were generated using a slightly modified version of the stepwise differentiation protocol of a previously published study (Kuwahara et al., 2015). Briefly, iPSCs were detached and dissociated into single cells using TrypLE Express (Gibco), and re-aggregated using low-cell-adhesion V-bottomed 96-well plates (Sumitomo Bakelite) in embryoid body (EB) medium (10,000 cells per well, 100 μL) containing 20 μM Y-27632 (Selleck) under 5% CO_2_ at 37°C. Recombinant human bone morphogenetic protein 4 (BPM4; R&D system, United States) was added to the culture at a final concentration of 55 ng/ml on differentiation day (D) 6 and its concentration was reduced by half when half of the medium was changed every third day. On D18, the aggregates were transferred to ultra-low adhesion 6-well plates (6 aggregates per well) in neural retinal differentiation medium containing DMEM/F12-Glutamax (Gibco), 1% N2 supplement (Gibco), 10% fetal bovine serum (FBS), 0.5 μM retinoic acid (Sigma), 0.1 mM taurine (Sigma), 0.25 mg/ml Fungizone (Gibco), 100U/mL penicillin and 100 mg/ml streptomycin under 40% O_2_ and 5% CO_2_. RPE cells were produced using directed differentiation protocol following the sequential use of 10 mM nicotinamide (Sigma), 100 ng/ml activin A (Peprotech) and 3 μM chir99021(Stemcell) (Regent et al., 2019).

### 2.2 RNA-sequencing analysis

Total RNA of 6-8 organoids at D18 were extracted from RPiPSCs and NiPSCs, and were submitted for library construction and sequencing using the BGISEQ-500 platform (BGI-Shenzhen, China). Three repetitions were performed for each group. RNA-seq raw data were initially filtered by removing adaptors, reads with more than 5% unknown bases and low-quality reads to obtain clean data after quality control. The clean data were then aligned to the human genome (GCF_000001405.39_GRCh38.p13) using the HISAT2 v2.0.4 software (Kim et al., 2015). The DEGseq package of R software (v3.1.1) was used to detect differential expression genes (DEGs) for gene expression analysis. The significance of DEGs was defined by the combination of the |fold change| ≥2 and Q-value <0.05 (Love et al., 2014).

### 2.3 Data-independent acquisition (DIA) proteomic analysis

DIA proteomic analyses were performed as previously described (Muntel et al., 2019). Briefly, 10 organoids at D34 were collected as one group, and three samples were set for each group. The DIA was subcontracted to Shanghai Omicsolution Co., Ltd. (Shanghai, China). Six samples of RP and control groups were processed individually by DIA to assess their proteome differences. MS1 and MS2 data were all acquired by random order. The iRT Kit (Ki3002, Biognosys AG) was used to calibrate the retention time of the extracted peptide peaks. Statistical assessment of the DIA dataset, including data normalization and relative protein quantification, was performed using Spectronaut 15 (Biognosys AG). Uniprot (https://www.uniprot.org) was used for retrieval of interacting genes and proteins and converted UniprotKB.ID of proteins into gene names. After DEGseq analysis, differently expressed proteins were filtered if the |fold change| ≥1.2 and Q-value <0.05.

### 2.4 Bioinformatic analysis

Principal component analysis (PCA) was performed separately on each dataset using the R function prcomp () from the stats package with default parameters. Hierarchical cluster analysis (HCA) is an algorithmic approach to identify discrete groups with varying degrees of similarity in a dataset represented by a similarity matrix. This analysis was processed using the pheatmap package (https://CRAN.R-project.org/package = pheatmap). A volcano plot is a type of scatter-plot that can quickly identify changes in large datasets by plotting significance versus fold-change on the y- and *x*-axes, respectively. It was drawn using the ggplot2 package (http://ggplot2.org). To take insight to the changes of phenotype, GO (http://www.geneontology.org/) and KEGG (https://www.kegg.jp/) enrichment analysis of annotated different expressed genes and proteins were performed by Phyper (https://en.wikipedia.org/wiki/Hypergeometric_distribution) based on Hypergeometric test. The significant levels of terms and pathways were selected with Q-value <0.05.

### 2.5 Real-time quantitative PCR (RT-qPCR)

RT-qPCR was used to determine the relative levels of gene transcription in organoid samples. Briefly, total RNA was extracted using the commercial RNAprep Pure Cell Kit (Tiangen) following the manufacturer’s protocol. Total RNA was quantified using a spectrophotometer, for which 1 μg of total RNA was reversely transcribed into cDNA using the ReverTra Ace qPCR RT Kit (Toyobo). RT-qPCR amplification and detection were performed using the SYBR Green Master Mix (Toyobo) on the LightCycler 480 real-time PCR system (Biorad). Relative gene expression levels were normalized to β-actin and calculated using the 2^−ΔΔCt^ method. Data are shown as means ± SD (*p < 0.05; **p < 0.01; ***p < 0.001; *n* = 3). The sequences of all primers are shown in Supplementary Table S1.

### 2.6 Western blotting

Fresh organoids were lysed in RIPA buffer (Thermo Fisher Scientific) and quantified using the BCA Protein Assay Kit (Thermo Fisher Scientific). Proteins were separated by SDS-PAGE, subjected to electrophoretic transfer on PVDF membranes (Merck Millipore), blocked and blotted with primary antibodies at 4°C overnight (rabbit anti-bcl2 antibody, Sigma; rabbit anti-bax antibody, Sigma; mouse anti-bestrophin (BEST1) antibody, Abcam; rabbit anti-melanoma gp100 (PMEL) antibody, Abcam; rabbit anti-β actin antibody, Bioss; rabbit anti-GAPDH antibody, Invitrogen). The blotting bands were incubated with secondary antibodies (HRP-labeled goat anti-rabbit antibody, CST; HRP-labeled goat anti-mouse antibody, CST) and illuminated by Immobilon Western Chemiluminescent HRP (Merck Millipore, Germany) and were captured using the chemiluminescencesystem (Biorad). Primary antibodies and secondary antibodies were diluted in Tris-Buffered Saline Tween-20 (TBST) buffer containing 5% (wt/vol) Bovine Serum Albumin (BSA). During incubations, PVDF membranes were washed with TBST buffer three times. The grayscale calculation of western blot images was performed by ImageJ software. GAPDH and β actin were used as an internal reference. The grayscale ratio of RP group to the control group was calculated. Data are representing as means ± SD (*p < 0.05; **p < 0.01; ***p < 0.001; *n* = 3). The experiments were performed with three replicates.

### 2.7 Immunofluorescence staining

Retinal organoids derived from RPiPSCs or NiPSCs were washed with PBS and rapidly frozen in an embedding medium (Sakura Finetek) before sectioning. The sections (10 μm) or RPE cells were thawed, air-dried, and fixed in 4% paraformaldehyde at room temperature for 15 min. Then, they were incubated with blocking buffer (3% BSA diluted in PBS) for 1 h, and primary antibodies for 2 h at room temperature. Next, they were incubated with a secondary fluorescent antibody (goat anti-mouse IgG H&L Alexa Fluor 488, Thermo fisher; goat anti-rabbit IgG H&L Alexa Fluor 594, Thermo fisher; donkey anti-goat IgG H&L Alexa Fluor 594, Thermo fisher) for 1 h at room temperature in the dark. The nuclei were stained with DAPI for 5 min. The sections were washed with PBS between these incubations. Finally, the sections were mounted with fluorescence mounting medium, and immunofluorescent images were captured with a fluorescence microscope (Leica). Detailed information of antibodies used are listed in Supplementary Table S2.

### 2.8 Terminal deoxynucleotidyl transferase dUTP nick-end labeling (TUNEL) staining

Retinal organoids were collected and prepared for cryosections. TUNEL kit (Beyotime Biotechnology) was suited for apoptosis analysis. Briefly, cryosections were fixed using 4% paraformaldehyde at room temperature for 30 min, then permeabilized in 1% Triton-X 100 for 15 min at 37°C. Then, sections were then incubated with TUNEL assay solution in the dark for 1 h at 37°C. The nuclei were stained with DAPI for 5 min. After washing three times with PBS, sections were mounted with fluorescence mounting medium and captured with a fluorescence microscope (Leica). ImageJ software was used to quantify DAPI-positive and TUNEL-positive areas. Data were shown as the percentage of TUNEL-positive areas.

### 2.9 Scanning electron microscopy (SEM)

The surface microstructures of RPE cells were captured using SEM. Samples were prepared as previously described (Cui et al., 2018). Briefly, the samples were fixed with 2.5% glutaraldehyde for 2 h, washed three times with PBS, dehydrated using a gradient concentration of ethanol (70, 80, 90, 100, 100, and 100%) for 10 min each, and coated with a gold-palladium alloy. They were then viewed using SEM on a JSM-T300-SEM instrument (JEOL Technics Co. Ltd.).

### 2.10 Fabrication of the microfluidic device

The microfluidic device was fabricated with a culture chamber of width 3.3 cm and height 2.45 cm. First, a mold was made using 3D printing based on the chamber size. Second, Polydimethylsiloxane (PDMS) Sygard 184 (Dow Corning) were mixed manually for 10 min at a weight ratio of 10:1. After degassing to remove entrapped bubbles, the mixture was poured into the mold and kept at 65°C for 2 h to solidify. Lastly, the PDMS culture chamber was carefully peeled off from the mold and arranged on a 9 cm culture dish. The fluidic inlet and outlet were created with a biopsy punch followed by testing the perfusion function with distilled water. The PDMS pieces were sterilized with 75% alcohol for 24 h, PBS for 2 h, and UV irradiation for 1 h. To promote adhesion, they were then precoated with 1% matrigel for 2 h. RPiPSCs derived RPE cells were seeded, and 2 days later, RPiPSCs derived ROs embedded with matrigel at D18 were placed on the RPE cells. The co-cultures were subjected to microfluidic systems for a total of 30 days. The flow rate of the aqueous phase (200 μL/min) was precisely controlled using a peristaltic pump (Longer).

### 2.11 Statistical analysis

All experiments were performed with at least 3 different iPSCs clones from patient and healthy controls. The data are represented as averages ± standard deviations. The unpaired two-tailed *t*-test was performed for comparison between groups using GraphPad 8.0 software. *p* values <0.05 were considered statistically significant. The fluorescence intensity, area calculation and grayscale analysis were performed using ImageJ (National Institutes of Health, Bethesda, MD, United States).

## 3 Results

### 3.1 Generation of ROs for disease modeling

**FIGURE 1:**
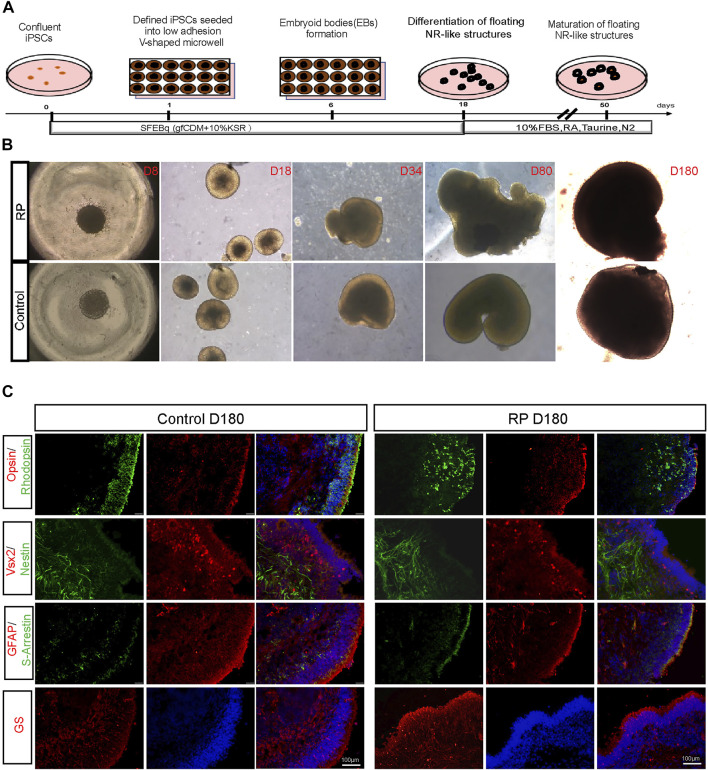
Generation of ROs derived from iPSCs with USH2A mutations. **(A)** Schematic representation of iPSCs differentiation along the ROs lineage. **(B)** Bright images of ROs at D8-D180 derived from RPiPSCs and NiPSCs with induction of BMP4, taurine and retinoic acid at different time points (×5). **(C)** Immunostaining images of cytosections of ROs were positive for markers of rod (rhodopsin), cone (opsin and S-arrestin), müller (GS), astrocyte (GFAP) and retinal progenitor cells (VSX2 and Nestin).

Human ROs were generated following the methods described in Figure 1A. Cells were sphere forming when cultured in suspension on a V-shaped bottom plate. During D8-D180, 3D organoids derived from iPSCs exhibited neural retina structures in both groups (Figure 1B). Immunostaining images showed that organoids were positive for VSX2 and nestin (retinal progenitor cell), opsin (cone), S-arrestin (cone) and rhodopsin (rod), glutamine synthetase (müller cell) and GFAP (astrocyte) (Figure 1C). To better understand the role of USH2A mutations involved in the pathogenesis of RP, we compared the expression levels of USH2A in patient-derived ROs with normal controls. Compared with the control group, fewer USH2A-positive areas were observed in the RP group (Supplementary Figure S2A-B). Besides, the data showed that ROs USH2A mRNA expression levels derived from both groups were not expressed until D80, and those of the patient group were lower than the controls (Supplementary Figure S2C). Altogether these data showed that we were able to establish an *in vitro* disease model of RP with mutated USH2A.

### 3.2 Association of USH2A mutations with abnormal RPE development

**FIGURE 2:**
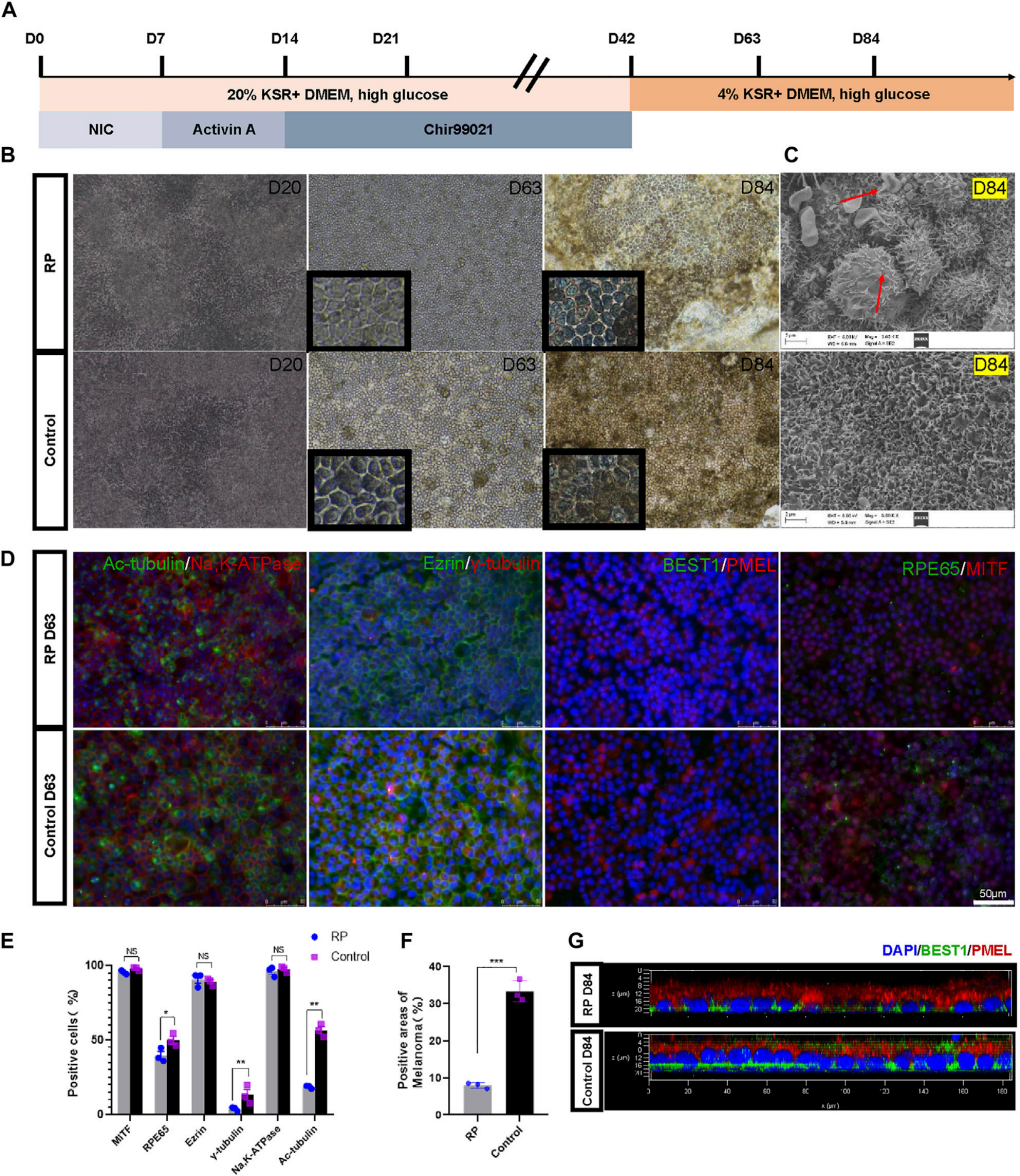
Directed differentiation protocol of RPE cells. **(A)** Schematic representation of the protocol. **(B)** Representative images of RPE cells at days 20, 63 and 84. **(C)** SEM images of RPE cells at D84. **(D)** Immunofluorescent staining of RPE specific markers. (Scale bar, 50 μm) **(E–F)** Positive cells or areas analysis. **(G)** Confocal micrographs of RPE cells stained with BEST1 (green) and PMEL (red). Data are shown as mean ± SD. *p < 0.05, **p < 0.01, ***p < 0.001, ns: no significance (*n* = 3).

Here, nicotinamide, activin A and Chir99021 were sequentially used to differentiate iPSCs into RPE (Figure 2A). During the first 20 days of culture, the cells had indistinct margins. By D63, the culture plates were covered with cobblestone-like and pigmented cells (Figure 2B). Scanning electron microscopy showed abnormal vacuoles of RPE cells derived from RPiPSCs at D84, indicating that cell death occurred in RPE cells with USH2A mutations (Figure 2C) (Zhang et al., 2018). RPE cells derived from both groups were greater than 95% expressing the transcription factor MITF. As an indicator of RPE cells maturity, we evaluated the apical-basal polarization of specific markers. As expected, RPE cells expressed the microvilli protein ezrin and the Na,K-ATPase pumps at their apical membrane without significant difference between the two groups. Cilium-related protein acetyl-α-tubulin (Ac-tubulin), basal body-related γ-tubulin and RPE65 protein markedly decreased in the RP group on D63. Immunofluorescence assay also showed that the percentage areas of PMEL were downregulated in RPiPSCs-RPE cells (Figures 2D–F). The calcium activated chloride channel, bestrophin (BEST1) was mainly localized at the baso-lateral compartment with reduced expression in the RP group on D84 (Figure 2G). Taken together, these findings suggest that USH2A mutation was associated with abnormal RPE development and abnormality in RPE65 protein expression, pigmentation and cilium organization.

### 3.3 Transcriptomics and proteomics analysis for early ROs with USH2A mutations

**FIGURE 3:**
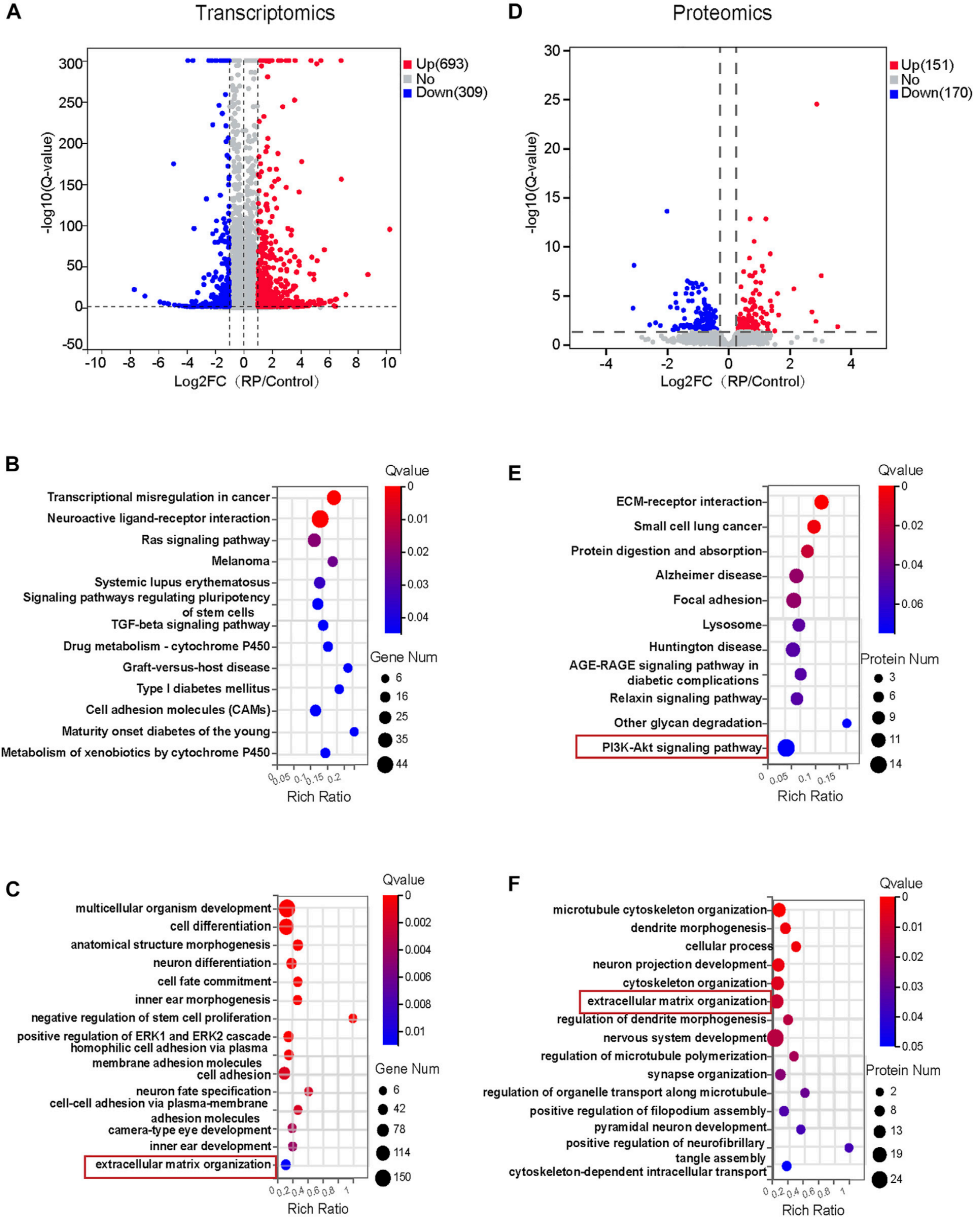
Transcriptomics and proteomics analysis for early ROs with USH2A mutations. **(A)** Volcano plot showing 693 DEGs were upregulated and 309 DEGs were downregulated in the RP group. **(B)** The 13 significantly changed signaling pathways based on DEGs in the RP group. **(C)**The 15 GO biological process terms based on DEGs. **(D)** Volcano plot showing 422 differentially expressed proteins, 151 proteins were upregulated and 170 proteins were downregulated. **(E)** The 11 significantly changed signaling pathways based on differentially expressed proteins. **(F)** The 15 GO biological process terms based on up-regulated and down-regulated proteins were selected.

To investigate the molecular mechanisms responsible for the biological effects of USH2A mutations, RNA-sequencing transcriptomics and DIA-based proteomics of both organoid groups were performed on D18 and D34, respectively. Pearson’s correlation coefficient, used to assess the correlation between the two groups of samples, indicated a strong correlation in each group of samples and the PCA showed that significant differences between the two groups of samples (Supplementary Figure S3). The expression of DEGs between the two groups was also compared. Volcano plots illustrated all transcripts (Figure 3A) and protein abundance (Figure 3D) in terms of fold change and Q-value. Transcriptomic analysis showed that 693 DEGs were upregulated while 309 DEGs were downregulated. Proteomic analysis showed that 151 differentially expressed proteins were upregulated while 170 were downregulated. Based on DEGs, the cell proliferation and apoptosis associated pathways including ras signaling pathway and TGF-beta signaling pathway, and signaling pathways regulating pluripotency of stem cells, cell adhesion molecules and neuroactive ligand-receptor interaction were significantly altered (Figure 3B). We identified several enriched biological pathways, including ECM-receptor interaction, focal adhesion, lysosome and PI3K-Akt signaling pathway in protein-pathway enrichment analysis (Figure 3E). Further, GO enrichment analysis of DEGs and differential expressed proteins demonstrated significant changes in multiple biological processes at transcriptome and proteome levels. For instance, extracellular matrix organization was regulated in both RNA- and protein biological processes analysis (Figures 3C,F). In addition, the regulated RNA-related GO terms were associated with cell adhesions, cell fate commitment, negative regulation of stem cell proliferation, anatomic structure morphogenesis, inner ear morphogenesis, inner ear and camera-type eye development (Figure 3C). The regulated protein-related GO terms were associated with cytoskeleton organization. These comprised of processes such as microtubule cytoskeleton organization, regulation of microtubule polymerization and cytoskeleton-dependent intracellular transport (Figure 3F). In summary, these data indicated that USH2A mutations affected multiple pathways and biological processes that were partly correlated at the transcriptome and proteome levels; suggesting a controlled interplay of gene expression and protein synthesis.

### 3.4 Association of USH2A mutations of iPSCs and ROs with apoptosis

**FIGURE 4:**
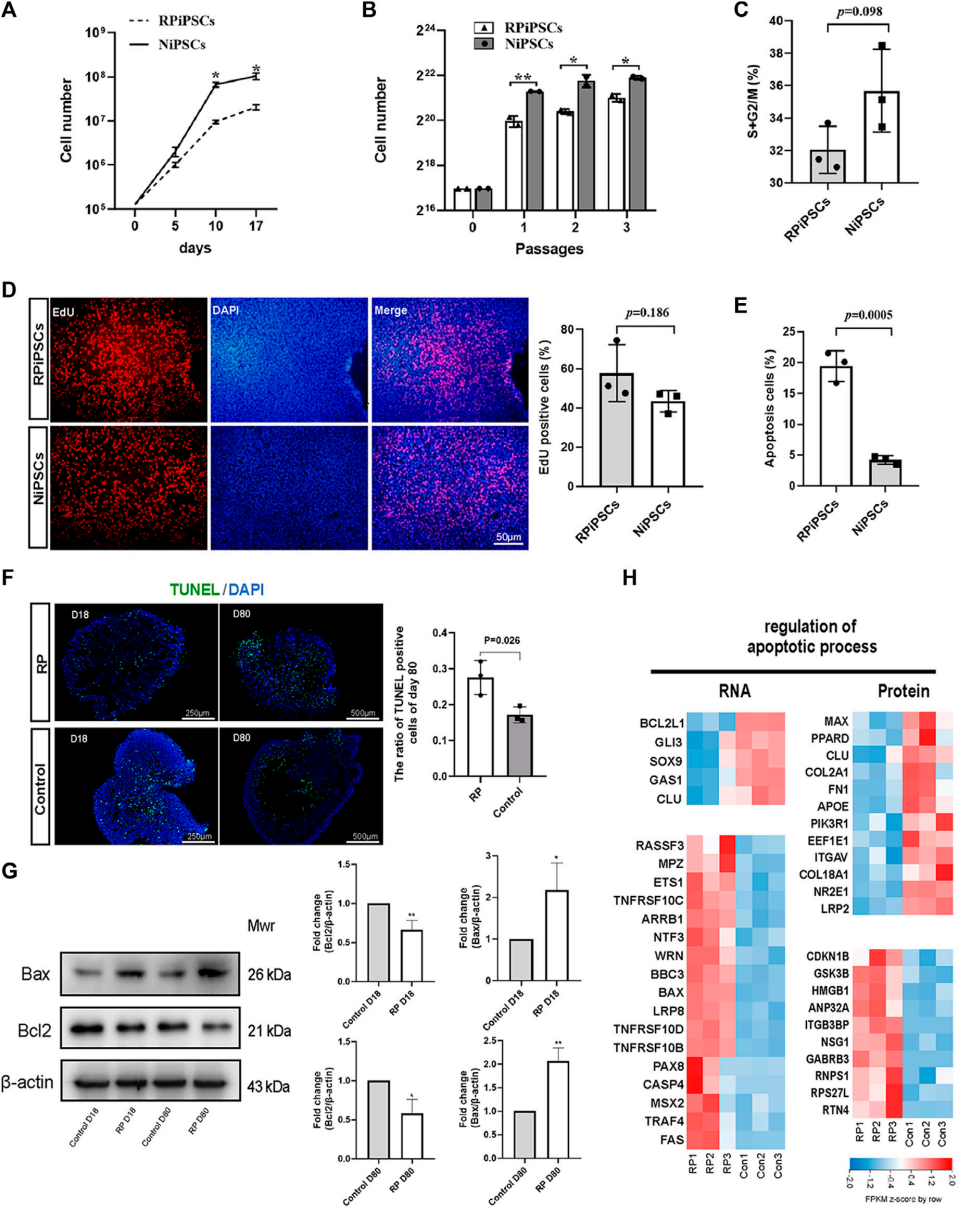
Increased apoptosis in iPSCs and ROs with USH2A variants **(A,B)** Cell numbers of iPSCs at different days and passages. **(C)** Cell cycle distribution analysis of two iPSC lines. **(D)** The percentage of EdU-positive cells in both iPSC lines. **(E)** Compared to control group, apoptosis rate of iPSCs significantly increased in the RP group. **(F)** Apoptotic cells were labeled with TUNEL (FITC, green). Nuclei are labeled with DAPI (blue) and the ratio of TUNEL-positive cells in retina organoids in both groups at D80. **(G)** Western blotting results of bcl2 and bax at D18 and D80. The grayscale ratio of RP group to the control group quantified in bar graphs. Data are shown as mean ± SD. *p < 0.05, **p < 0.01, ***p < 0.001 (*n* = 3). **(H)** Heatmap of gene expression changes and proteins related to regulation of apoptotic process. Upregulated and downregulation expression are in red or blue respectively. Bar is representing Z-score. Con: control.

When iPSCs were expanded under identical culture conditions, the number of RPiPSCs was found to be lower than the control group (Figures 4A,B). No significant difference in the cell cycle (Figure 4C; Supplementary Figure S4A) or proliferation (Figure 4D) between the two groups was observed. Flow cytometry results demonstrated that the percentage of apoptotic cells was significantly increased in the RP group, compared with the NiPSCs (Figure 4E; Supplementary Figure S4B). Collectively, the RPiPSCs clones demonstrated relatively slower growth due to increased cell apoptosis.

Next, we assessed the apoptosis of ROs using the TUNEL assay on D18 and D80. We observed that the nuclei of TUNEL-positive (apoptotic) cells stained green, and the percentage of TUNEL-positive cells was higher in the RP groups on D80 (Figure 4F). Further, western blot experiments revealed that the expression level of anti-apoptotic protein BCL2 was decreased in ROs with USH2A mutations. In contrast, pro-apoptotic protein BAX was increased on D18 and D80 (Figure 4G). According to transcriptional and proteomic results, multiple genes and proteins related to the apoptotic process were regulated in the RP groups (Figure 4H). For instance, anti-apoptotic related molecules such as BCL2L1 and CLU were downregulated at transcript levels in the RP groups. Conversely, pro-apoptotic associated factors, including tumor necrosis factor (TNF) receptor superfamily members (TNFRSF10B, TNFRSF10C and TNFRSF10D), FAS and BAX, were upregulated. Additionally, the ratio of apoptotic cells was elevated in the XT142-iPSCs line, compared with the NiPSCs (Supplementary Figure S5B). The ROs derived from XT142-iPSCs showed increased apoptosis including the higher percentage of TUNEL-positive cells and mRNA expression of BAX as well as the lower mRNA expression of BCL2 on D18 (Supplementary Figures S5C, S6). Thus, the above findings suggest that USH2A mutation could induce the apoptosis of iPSCs and ROs.

### 3.5 ROs with USH2A mutations showed abnormal ECM organization functions

**FIGURE 5:**
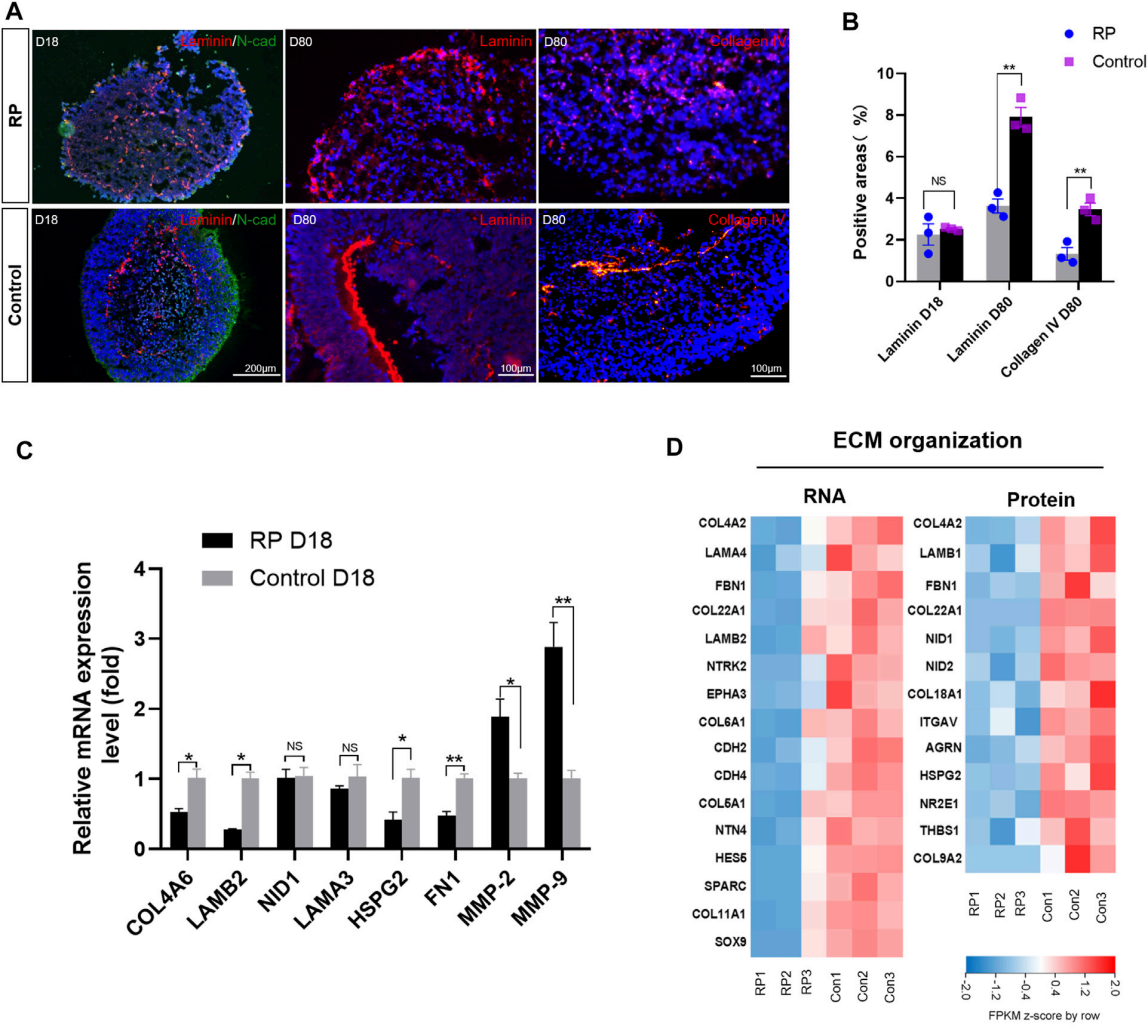
RPiPSCs derived ROs showing abnormal ECM organization functions. **(A)** Immunostaining of laminin (red) and collagen IV (red) of ROs in both groups. **(B)** Quantification of positive areas with laminin and collagen IV. **(C)** RT-qPCR results showing the expression of ECM-related molecules. Data are shown as mean ± SD. *p < 0.05, **p < 0.01, ***p < 0.001, ns: no significance (*n* = 3). **(D)** Heatmap of gene and protein expression changes related to ECM organization of ROs at D18. Upregulated and downregulation expression are in red or blue respectively. Bar is representing Z-score. Con: control.

To better understand the role of USH2A mutations in the ECM organization process of RP, ECM-related molecules were examined. Immunofluorescence results revealed that the structure of laminin was disorganized on D18, and its expression at the protein level was decreased in the RP group on D80 (Figure 5A). Similar results were found for collagen IV on D80 (Figures 5A,B). Moreover, RT-qPCR data showed that ECM components COL4A, LAMB2 and fibronectin (FN1) were downregulated on D18, while matrix-metalloproteinase (MMP)-2 and MMP9 were significantly increased in the retina organoids derived from RPiPSCs (Figure 5C). Bioinformatics data showed that ECM molecules, namely COL4A2, LAM, FBN1 and COL22A1, decreased markedly at both mRNA and protein expression levels in the RP groups. Besides, proteins encoded by NID1, NID2, AGRN, COL18A1, HSPG2 and ITGAV were at lower expression levels in the RP groups than in the normal group (Figure 5D). In addition, RT-qPCR results revealed an increased mRNA expression level of ECM-related genes for ROs in XT142-iPSCs group on D18 in comparison to the control group (Supplementary Figure S6). Based on our findings in the expression level of mRNA and proteins, we suggest that ROs with USH2Amutations could be related to abnormal ECM organization function.

### 3.6 Enhancement of ECM components formation in ROs and RPE on a microfluidic chip

**FIGURE 6:**
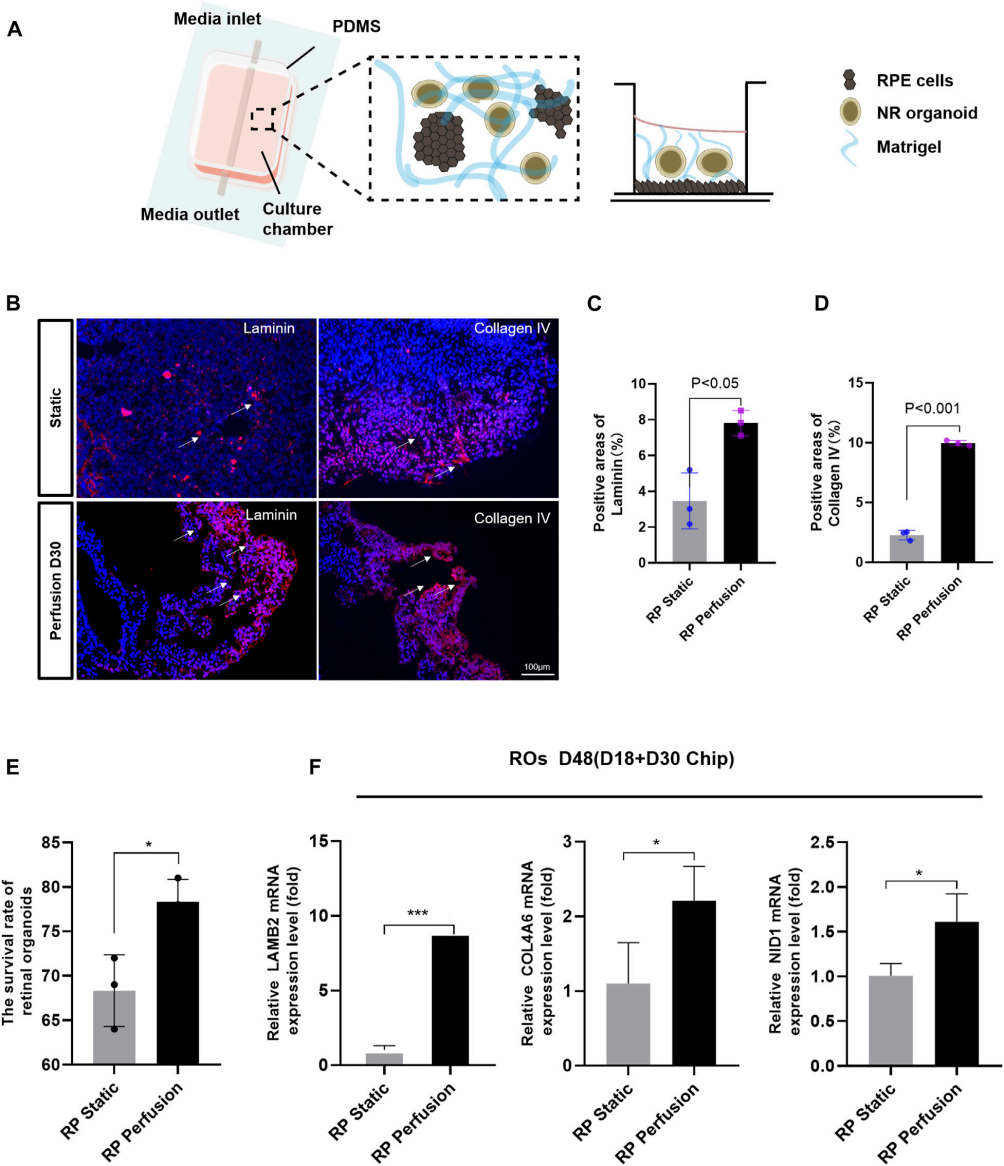
Microfluidic chip of perfusable ROs in co-culture with RPE. **(A)** Schematic of the microfluidic system. **(B)** Immunostaining images of laminin (red) and collagen IV (red) of ROs in response to perfusion with 200 μL/min after 30 days compared to static control condition. **(C,D)** Measurements of positive areas with laminin and collagen IV. **(E)** The survival rate of ROs. **(F)** After 30 days perfusion, RT-qPCR results show COL4A6, LAMB2 and NID1 mRNA expression levels of ROs. Data are shown as mean ± SD. *p < 0.05, **p < 0.01, ***p < 0.001 (*n* = 3). Scale bar:100 μm.

**FIGURE 7:**
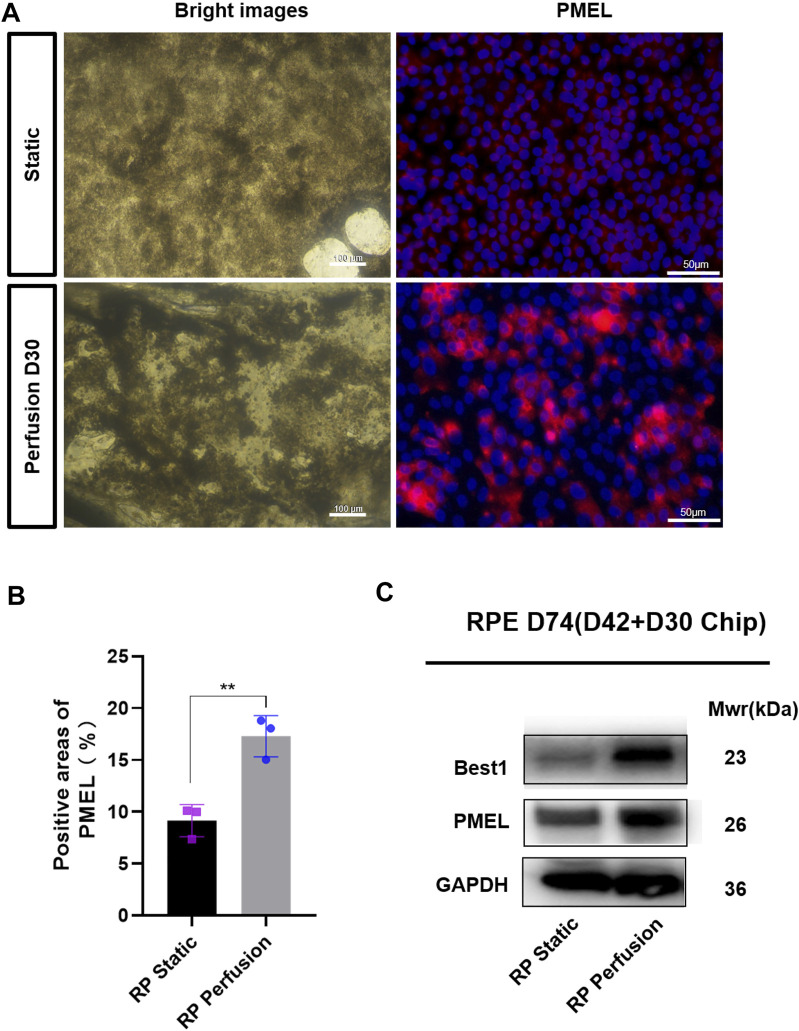
Quantitative comparison of RPE cells in two culture methods. **(A)** Bright images of RPE cells under two culture conditions and immunostaining images of PMEL (red). **(B)** Positive areas analysis. Data are shown as mean ± SD. **p < 0.01 (*n* = 3) **(C)** Perfused culture of RPE cells leads to an upregulation of BEST1 and PMEL. Scale bar:50 μm.

The microfluidic chip was designed to co-culture ROs with RPE cells and optimize the culture conditions (Figure 6A). Briefly, ROs derived from RPiPSCs on D18 were embedded with matrigel, placed on the RPiPSC-derived RPE cells, and subjected to microfluidic systems for 30 days. Both ROs and RPE showed normal morphology. We found that fewer ROs with USH2A mutations underwent apoptosis on the perfused chip, compared to static conditions (Figure 6E). Collagen IV and laminin positive staining significantly increased on the perfused chip (Figures 6B–D). Moreover, data from RT-qPCR experiments validated that COL4A6, LAMB2 and NID1 mRNA expression levels of ROs were higher in the perfusion groups (Figure 6F), demonstrating good agreement with the immunofluorescence staining results. On the other hand, we found that RPE cells derived from RPiPSCs were more pigmented under perfused conditions. Further, perfused culture was associated with an upregulation of PMEL (Figures 7A,B). Western blot results of PMEL were highly correlated with immunofluorescent analysis. BEST1 expressed in the basolateral membrane of RPE was also upregulated after 30 days of perfusion (Figure 7C). These findings showed that the microfluidic chip could stimulate ROs with USH2A mutations to produce ECM components and facilitate ROs and RPE development compared with static culture.

## 4 Discussion

**FIGURE 8:**
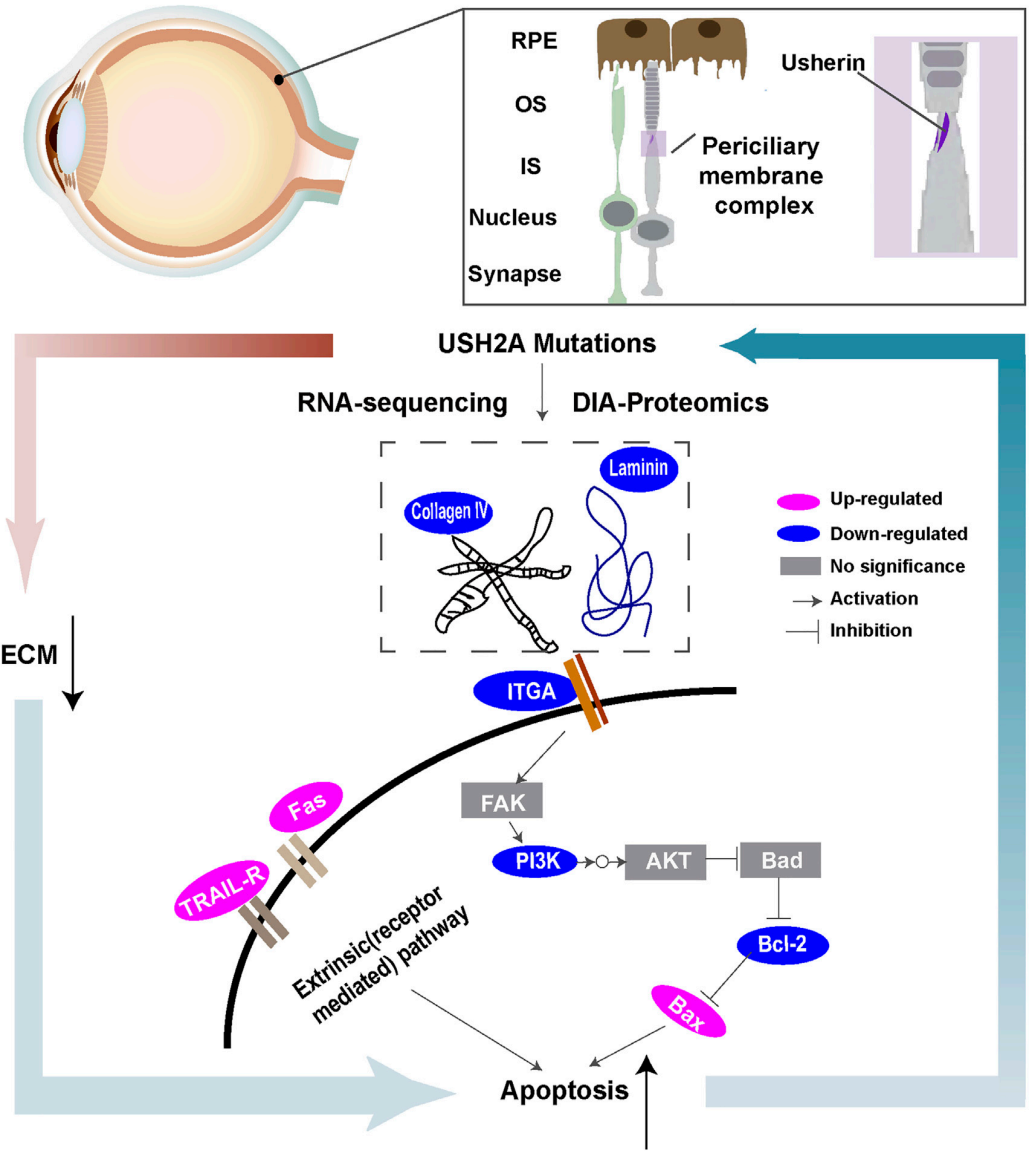
Schematic overview of ECM-receptor interactions of molecules with special emphasis on signaling pathways based on transcriptional and proteomic profiles.

RP is the most common inherited retinal dystrophy. Owing to its significant clinical and genetic heterogeneity, effective treatment options remain limited. Therefore, it is important to understand the pathogenesis of RP to develop efficacious therapies. In this study, a disease model derived from a RP patient with USH2A mutations was created. The main study findings were as follows. First, we observed higher apoptosis rates in iPSCs with USH2A mutations and their derived ROs. Second, based on the differentially expressed proteins from the KEGG database, signaling pathway diagrams associated with ECM-receptor interactions were plotted (Figure 8). Decreased levels of ECM constituents (laminin and collagen IV) were found to affect the interactions with their receptors (integrin αⅤ) and downregulated the PI3K-Akt signaling pathway. We suggested that a close relationship between USH2A mutations and defective ECM functions leading to downregulation of PI3K-Akt signaling pathway played an important role in cell apoptosis. Third, for the first time, we showed that microfluidic chip facilitated RPiPSCs derived ROs and RPE development, and could effectively upregulate ECM components such as laminin, collagen IV and nidogen under perfusion conditions.

In mammalian retinal photoreceptors, usherin is localized at the periciliary membrane complex. It is an ECM protein composed of laminin and fibronectin domains (Liu et al., 2007). The ECM is a major component of the cellular microenvironment, and its constituents have versatile roles during retinal development, including cellular proliferation, differentiation, migration, adhesion, maturation and axonal growth (Kundu et al., 2016; Dorgau et al., 2019). Recent studies in zebrafish models found that loss of usherin led to the aberrant assembly of fibronectin, causing defects in supporting photoreceptors and maintaining normal photoreceptor structure (Dona et al., 2018; Han et al., 2018). It is reported that the laminin network is assembled by the interaction of cell surface receptors which trigger the deposition of nidogen and perlecan proteins encoded by NID and HSPG2 respectively, which participate in cross-linking collagen IV network to generate the core of basement membranes (Lopez-Luppo et al., 2017). However, the ECM can be targeted and proteolytically cleaved by activating key members of the MMP family, leading to retinal degenerative diseases. For instance, laminin degradation by MMP9 was shown to promote ketamine-induced neuronal apoptosis in the early stage of rats’ diseased retina (Wu et al., 2020). These findings were consistent with this study’s results. We found that abnormal ECM organization was observed in ROs with USH2A mutations, which included decreased ECM components, such as laminin, collagen IV and fibronectin, and increased MMPs expression in the RP group. As an important cellular adhesion molecule, ITGAV (integrin αⅤ), involved in mediating the attachment of cells to the ECM, was also downregulated in the RP group. Interestingly, we also found that USH2A in ROs exhibited a lower gene expression in the patient group at D80. Such changes may be related to the development of photoreceptors in ROs. Our previous transcriptional study revealed that photoreceptor cell-related transcription factors were highly expressed from D80 to D136 in human ROs (Cui et al., 2020). In addition, usherin was found in a region between the outer and inner segments of the photoreceptors in mice retina (Liu et al., 2007). However, in our study, the low expression of USH2A without significant differences in both groups in the early stages of ROs could not account for the observed ECM disorders and abnormal ROs, and needs to be further explored in future studies.

We further investigated the downstream effects of ECM organization in RP by integrating transcriptomic and proteomic analysis. We observed that the downregulated proteins were mostly involved in the PI3K-Akt signaling pathway. Lin et al. observed that deactivating of the PI3K/mTOR pathway in the retina of rd10 mouse induced cone cell apoptosis *in vitro* (Lin et al., 2018). Based on these findings, we presumed that USH2A mutations led to decreased levels of ECM components and their receptors (integrin αⅤ) of ROs, and the abnormal interactions might negatively regulate the PI3K-Akt signaling pathway.

This study also revealed that iPSCs and ROs with USH2A mutations were associated with a high percentage of apoptotic cells. Additionally, a significant increase in the expression of pro-apoptotic related molecules such as bax, TNF, ras, HMGB1 and TIAM1 were confirmed by western blotting, RNA sequencing and DIA proteomic analysis that connected with abnormal ROs with USH2A mutations. Structural modeling analysis showed that USH2A pathogenic mutations were enriched in most laminin-related domains, specific FN3 domains, F16-F17 linker, transmembrane domain, and intracellular regions, suggesting that these usherin regions were functionally important (Yu et al., 2020). The mutation sites (c.8559–2A>G and c.9127_9129delTCC) of the RPiPSCs were assessed and located in FN3 motifs. Based on existing literature, apoptosis has been the predominant mechanism in retinal degeneration diseases (Olivares-González et al., 2021). Crucially, apoptosis can also be activated extrinsically following interaction between ligands produced by immune cells and death receptors (i.e., TNF or Fas) on the surface of damaged cells (Wajant, 2017). Several mechanisms, including the accumulation of aberrant ECM assembly and protein misfolding leading to ER stress, have been linked to triggering the death of rod photoreceptors of RP with USH2A mutations via some pathways (Tucker et al., 2013; Han et al., 2018). Based on the above findings, we suggest that insufficient ECM of retinal organoids with USH2A mutations triggered apoptosis by deactivating the PI3K-Akt signaling pathway.

Research studies relying on simplified static culture cannot maintain long-term culture due to lack of oxygen and nutrient diffusion to the cells, leading to the organoids’ necrosis (Zhang et al., 2021). Microfluidic technology offers the opportunity to accurately control fluid velocity and cell consumption and can mimic complex functional characteristics of human tissues such as inter-tissues crosstalk. It has been used in many research aspects extending from gut, liver, brain to cancer, but its application within retinal tissue research has been limited (Kim et al., 2016; Shirure et al., 2018; Wang et al., 2018; Peters et al., 2020). A pioneering study using microfluidic platforms showed that ROs could successfully establish *in vivo*-like physiological processes, including shed-out outer segments phagocytosis and calcium dynamics (Achberger et al., 2019). However, there has been few studies investigating ROs derived from patients on a microfluidic chip. Here, we co-cultured ROs and RPE derived from a patient diagnosed with RP in an adapted microfluidic chip. Compared with those maintained in static culture, the microfluidic platform facilitated ROs culture by upregulating ECM components. This result is consistent with other reports, showing an intestine-on-chip enhanced ECM remodeling of the stroma, basement membrane production, and speeding of epithelial differentiation (De Gregorio et al., 2020). RPE cells under perfusion were more pigmented and mature than in static conditions, highlighting the importance of perfusion culture for inducing physiological phenotype in RPE cells. Taken together, the microfluidic chip could facilitate ROs and RPE development.

In summary, this study revealed that USH2A mutations were associated with decreased ECM and increased apoptosis of ROs. By an integrated analysis of transcriptomics and proteomics at the ROs, we supposed that ROs with USH2A mutations reduced ECM-receptor interactions and deactivated the downstream signaling of the PI3K-Akt pathway triggering cell apoptosis, which might be the mechanism underlying the pathogenesis of RP with USH2A mutations. Our findings have drawn from three colonies of one patient caused by USH2A mutations (c.8559–2A>G and c.9127_9129delTCC) (KLRMMEi001-A) due to the low incidence (in particular, heterozygous mutations, one of which is a new mutation that we found). For further validation, an iPSCs line named XT142-iPS with USH2A gene mutation (only c.8559–2A>G) (KLRMMEi002-A) from a USH2 patient was created and used for ROs differentiation. We observed increased apoptosis and abnormal ECM organization functions of ROs derived from XT142-iPSCs, which accorded with the results of RPiPSCs (c.8559–2A>G and c.9127_9129delTCC). In future study, we will screen for more cases harboring the same mutations to confirm the findings described herein. Additionally, it should be noted that further studies validating the participation of the proposed signaling pathway is needed. Moreover, we present a retina-on-a-chip device design that co-cultures RP specific retinal organoids and RPE cells from iPSCs. The preliminary investigation was quite promising in terms of enhancement of ROs and RPE cells development. As a microfluid chip, the system with automated and accurate liquid handling demonstrated promising usefulness as a tool for studying neurodegenerative diseases. In the future, we expect to generate a vascularized retinal organoid-on-chip device to create an *in vivo*-like microenvironment that could better modeling the RP disease.

## Data Availability

The raw data supporting the conclusions of this article will be made available by the authors, without undue reservation.
