# Supplementary material for: Retinal organoids and microfluidic chip-based approaches to explore the retinitis pigmentosa with USH2A mutations
Source: Front Bioeng Biotechnol. 2022 Sep 14;10:939774. doi: 10.3389/fbioe.2022.939774 (PMC9524156; doi:10.3389/fbioe.2022.939774)
Supplement: Supplementary file 1 [file DataSheet1.docx]

Supplementary Material

# Supplementary Methods

# 5-Ethynyl-2’-deoxyuridine (EdU) assay

IPSCs were incubated with 50 μM EdU reagent (RiboBio, China) for 2 hours for EdU incorporation during DNA synthesis. Next, cell nucleus was stained with DAPI. The fluorescence images were captured under a fluorescence microscope (Leica).

**1.2 Flow cytometry**

The apoptotic percentage of iPSCs was analyzed by fluorescein isothiocyanate (FITC) and propidium iodide (PI) double-staining using an Apoptosis Detection Kit (Multi Sciences, China). Briefly, iPSCs were collected, washed with PBS and suspended in 100μL binding buffer. 10μL Annexin V-FITC and PI were then added and incubated for 15 minutes at room temperature in the dark. Cell samples were loaded onto the FACS cytometer (BD Biosciences), and cell apoptotic rate was calculated by BD FACS software.

# Supplementary Figures and Tables

## Supplementary Figures


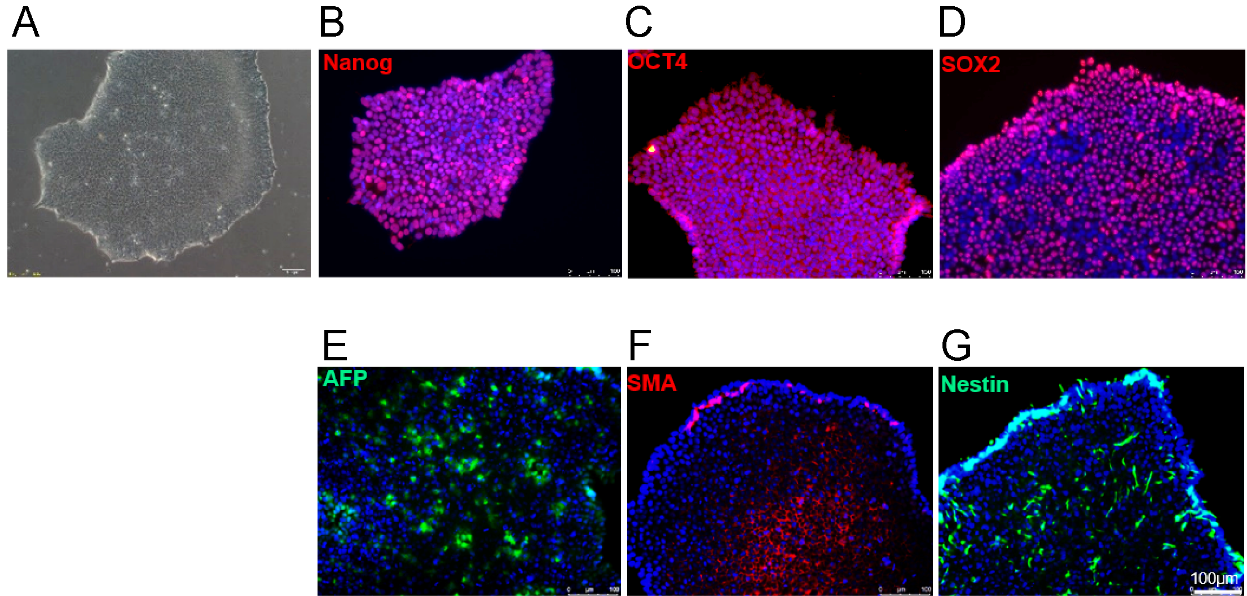


**Supplementary Figure 1.** Characteristics of iPSCs derived from RP patient. (A) Morphology of RP patient-specific iPSCs. (B-D) Immunofluorescence staining of the pluripotency markers Nanog, OCT4 and SOX2. (E-G) Positive staining of AFP, SMA and Nestin showing the capacity to form three germ layers in the RP patient-specific iPSCs. (Scale bars:100μm)


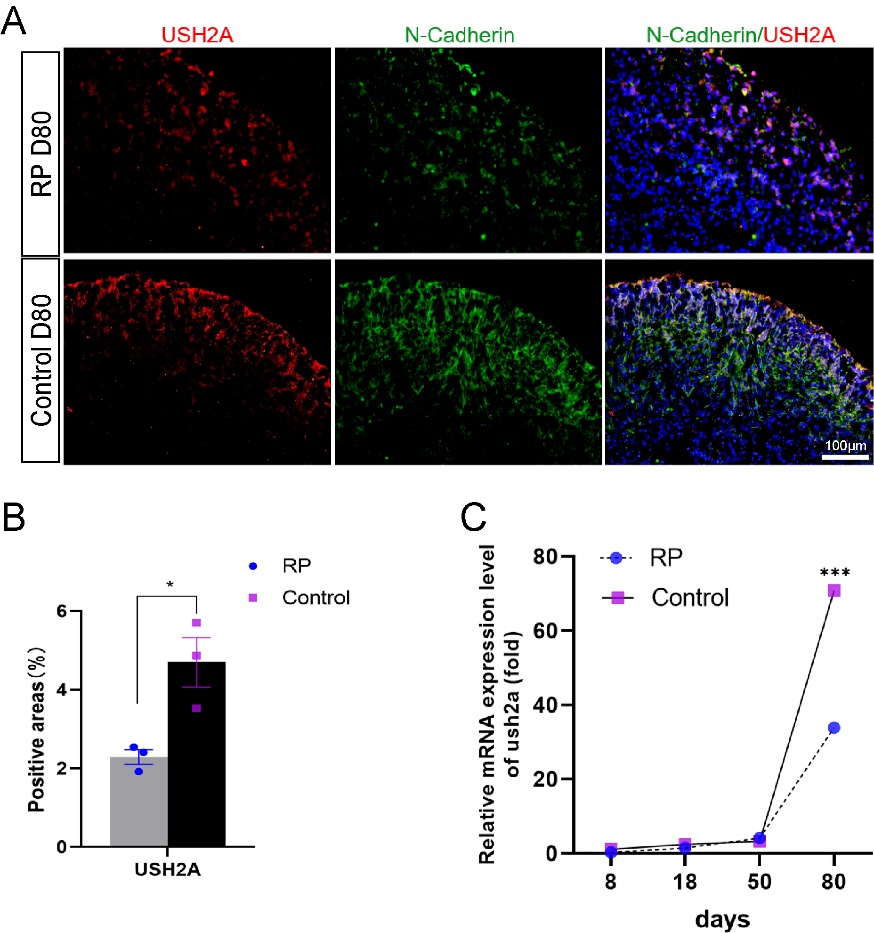


**Supplementary Figure 2.** USH2A expressions of ROs. (A) Immunostaining images of ROs were positive for USH2A (red) in both groups. N-cadherin (green) was an apical marker for ROs. (B) Quantification of positive areas stained with USH2A. (C) RT-qPCR analysis reveals USH2A mRNA expression at day 8- 50 in RP-organoids and normal controls. Data are shown as mean±SD. *p<0.05, **p<0.01, ***p<0.001 (n=3). Scale bars:100μm


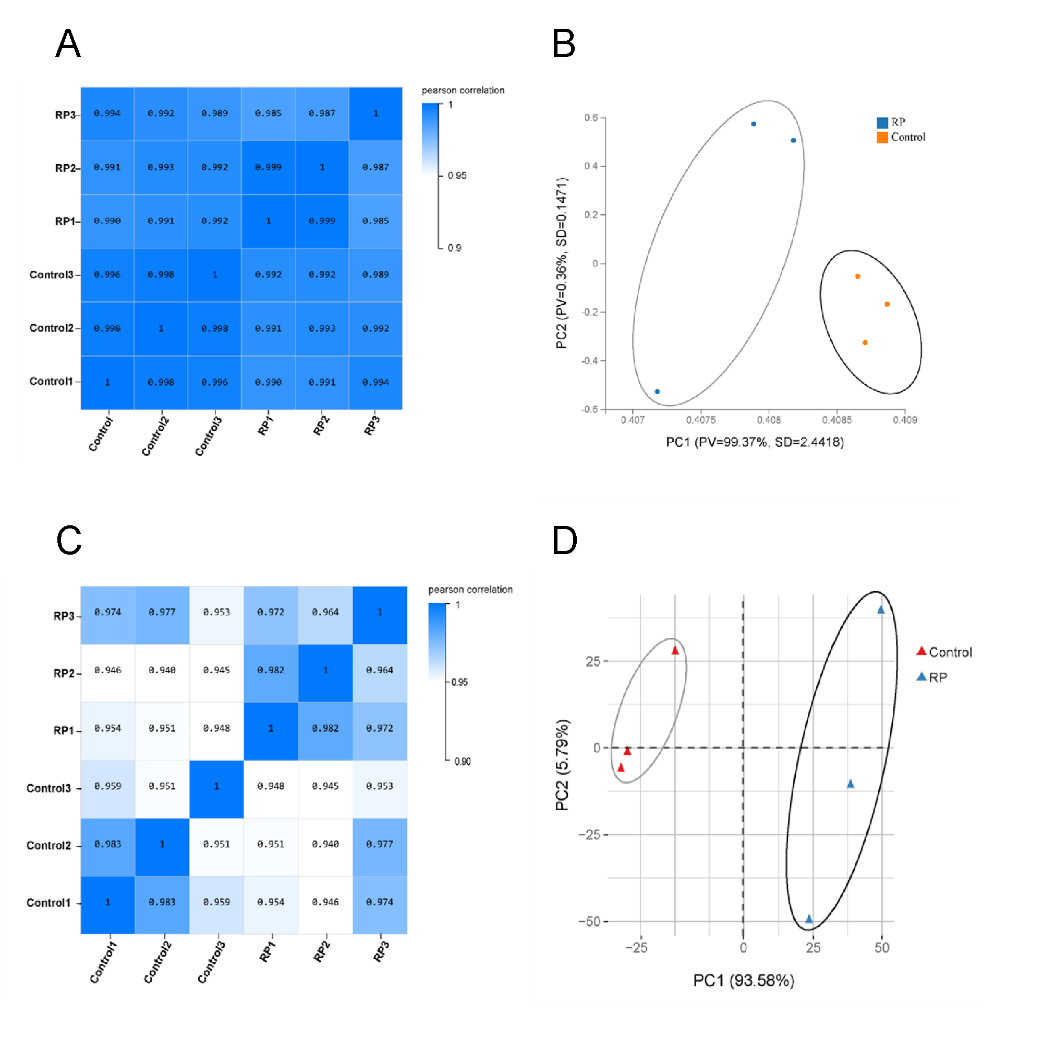


**Supplementary Figure 3.** Transcriptomics and proteomics data showing the correlation and differences between the two groups of samples. (A) The Pearson heatmap showing the correlation between the two groups of samples. (B) The PCA plots for RNA sequencing samples showing significant differences between two groups of samples. (C) Heatmap of Pearson’s correlation coefficients. (D) The PCA of protein expression data from samples of two groups indicating differences of the two groups.


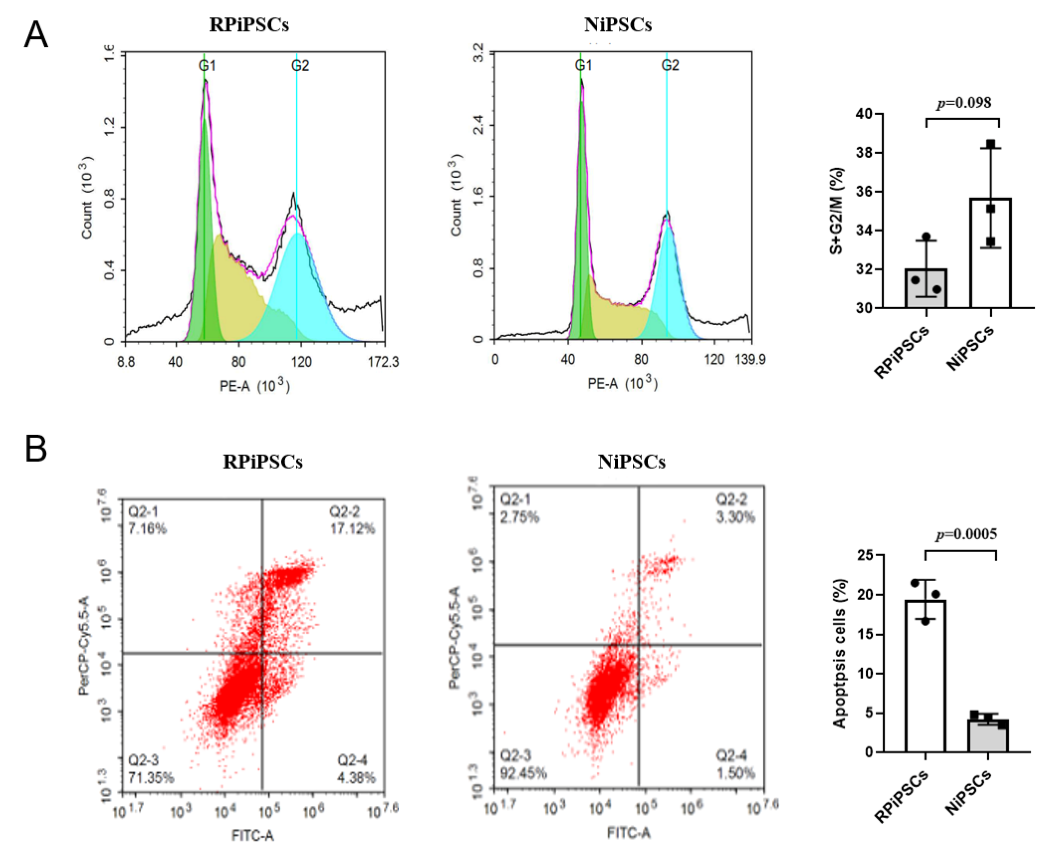


**Supplementary Figure 4.** (A) Cell cycle distribution analysis of RPiPSCs and NiPSCs. (B) Flow cytometry analysis of cell apoptosis between RPiPSCs and NiPSCs.


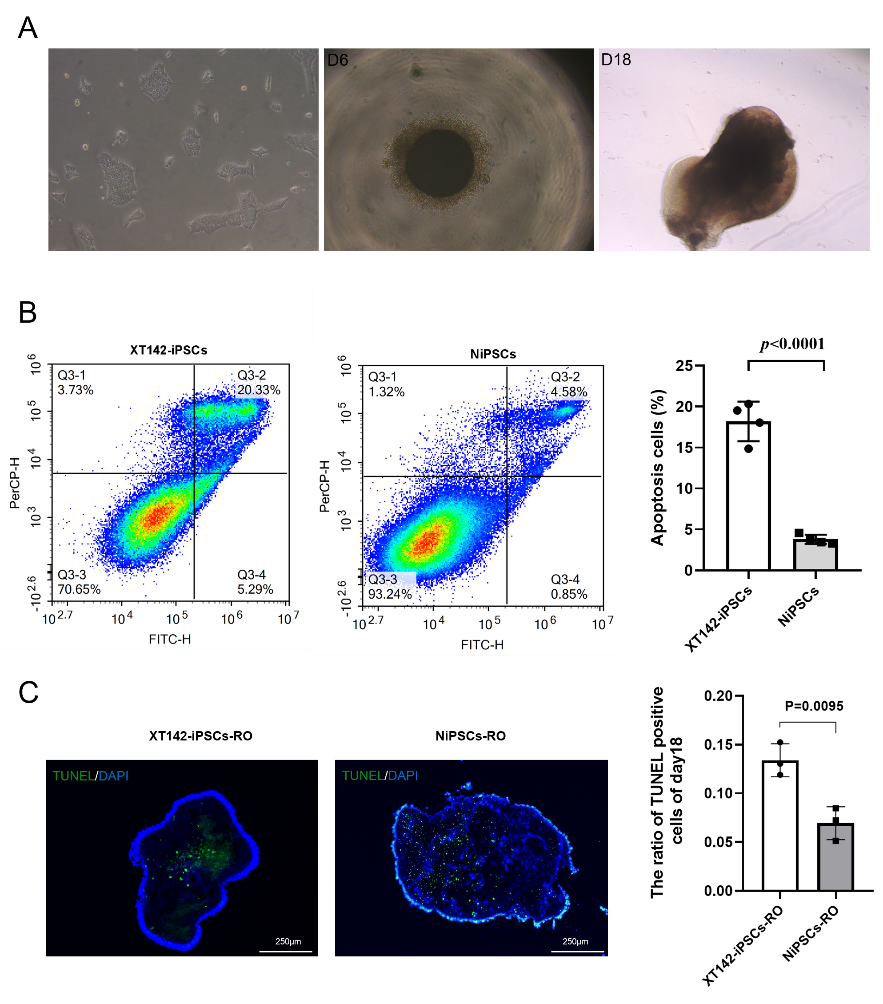


**Supplementary Figure 5.** XT142-iPSCs and derived ROs showing increased apoptosis. (A) Bright images of XT142-iPSCs and derived ROs at D6 and D18 (5×). (B) Compared to control group, apoptosis rate of XT142-iPSCs significantly increased. (C) The ratio of TUNEL-positive cells in ROs derived from XT142-iPSCs was higher than the control group at D18. Apoptotic cells were labeled with TUNEL (FITC, green), nuclei are labeled with DAPI (blue). Data are shown as mean±SD (n=3), The statistical analysis was performed using an unpaired *t*-test. Scale bar:250μm


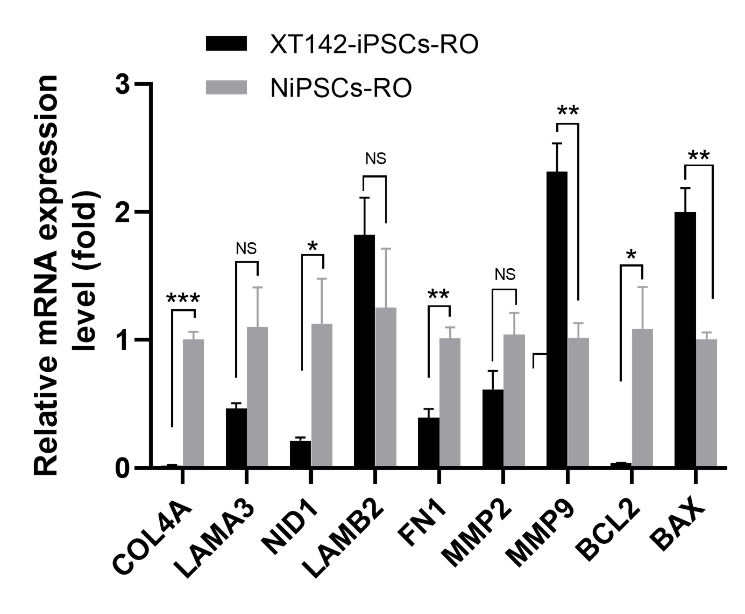


**Supplementary Figure 6.** ECM- and apoptotic genes mRNA levels in ROs derived from XT142-iPSCs and NiPSCs. ROs in both groups were collected for total RNA isolation. RT-PCR was conducted to quantify the mRNA expression levels of COL4A, LAMA3, NID1, LAMB2, FN1, MMP2, MMP9, BCL2 and BAX. Data were analyzed from 3 independent samples and represented as means±SD. The statistical analysis was performed using an unpaired *t*-test (*p<0.05; **p<0.01;***p<0.001; NS: no significance).

Supplementary Tables

**Table1. List of primers**

| Gene | Forward Primer （5’-3’） | Reverse Primer （5’-3’） |
| --- | --- | --- |
| MMP2 | ATTGTATTTGATGGCATCGCTC | ATTCATTCCCTGCAAAGAACAC |
| MMP9 | CAGTACCGAGAGAAAGCCTATT | CAGGATGTCATAGGTCACGTAG |
| USH2A | CAAACAGCAGTGCTTGGGTA | TGCGGAAGTCACATTGGTTA |
| LAMB2 | AAATCTTGTGCTTGCAATCCTC | CGCAAAGCAACTGTTGTTTAAG |
| LAMA3 | CTGCAGTTTAAACAAACCACCT | CAGCTGGTTGATACGAAAAGTC |
| COL4A6 | GGGTGTGAAGAAGTTTGATGTG | CCTTTCACCCTTGTCTCCTTTA |
| NID1 | CTTTTGGTGCAATCAACCAAAC | CATTATACAAGGCAAAGACCCG |
| FN1 | AATAGATGCAACGATCAGGACA | GCAGGTTTCCTCGATTATCCTT |
| HSPG2 | CTACTTCTATTGGTCCCGTGAG | ATTACGGCAGGTGCAAATGTAG |
| BCL2 | GACTTCGCCGAGATGTCCAG | GAACTCAAAGAAGGCCACAATC |
| BAX | CGAACTGGACAGTAACATGGAG | CAGTTTGCTGGCAAAGTAGAAA |
| β-actin | CTCCATCCTGGCCTCGCTGT | GCTGTCACCTTCACCGTTCC |

**Table2.** Primary Antibodies Used for Immunofluorescence Staining and Western Blot

| Antigen | host | Dilution Ratio | Catalog Number | Supplier |
| --- | --- | --- | --- | --- |
| Opsin | rabbit | 1/50 | Sc-30022 | Santa cruz |
| Rhodopsin | mouse | 1/50 | Sc-57432 | Santa cruz |
| Vsx2 | rabbit | 1/200 | 25852-1-AP | Proteintech |
| GFAP | rabbit | 1/1000 | NB300-141 | Novusbio |
| S-arrestin | mouse | 1/200 | NB100-2385 | Novusbio |
| GS | rabbit | 1/200 | DF7341 | Affinity |
| Acetylated-tubulin | mouse | 1/500 | T7451 | Sigma |
| Na,K ATPase | rabbit | 1/100 | 23565S | CST |
| Ezrin | mouse | 1/100 | Sc58758 | Santa cruz |
| γ-tubulin | rabbit | 1/1000 | T3559 | Sigma |
| BEST1 | mouse | 1/100 | Ab2182 | Abcam |
| PMEL | rabbit | 1/100 | Ab137078 | Abcam |
| RPE65 | mouse | 1/100 | Ab78036 | Abcam |
| MiTF | rabbit | 1/1000 | Ab3201 | Abcam |
| N-cadherin | mouse | 1:250 | 610921 | BD |
| Laminin | rabbit | 1:100 | Ab11575 | Abcam |
| Collagen IV | goat | 1:200 | AB769 | Sigma |
| Nanog | rabbit | 1:100 | 14295-1 | Proteintech |
| OCT4 | rabbit | 1:200 | 2750S | CST |
| SOX2 | rabbit | 1:400 | 3579S | CST |
| AFP | mouse | 1:200 | MA5-14666 | Invitrogen |
| SMA | rabbit | 1:50 | Bs-0189R | Bioss |
| Nestin | mouse | 1:50 | Sc-23927 | Santa Cruz |
| USH2A | rabbit | 1:200 | Ab82714 | Abcam |
| BCL2 | rabbit | 1:1000 | SAB4500003 | Sigma |
| BAX | rabbit | 1:1000 | SAB5700002 | Sigma |
